# Increased Belching After Sleeve Gastrectomy

**DOI:** 10.1007/s11695-015-1718-3

**Published:** 2015-06-23

**Authors:** Jan S. Burgerhart, Paul C. van de Meeberg, Femke A. Mauritz, Erik J. Schoon, Johannes F. Smulders, Peter D. Siersema, André J. P. M. Smout

**Affiliations:** Department of Gastroenterology and Hepatology, University Medical Center Utrecht, Utrecht, The Netherlands; Dutch Obesity Clinic, Hilversum, The Netherlands; Department of Gastroenterology and Hepatology, Slingeland Hospital, Doetinchem, The Netherlands; Department of Gastroenterology and Hepatology, Catharina Hospital, Eindhoven, The Netherlands; Department of Surgery, Catharina Hospital, Eindhoven, The Netherlands; Department of Gastroenterology and Hepatology, Academic Medical Center, Amsterdam, The Netherlands

**Keywords:** Sleeve gastrectomy, Belching, Impedance monitoring, Reflux, Swallows

## Abstract

**Introduction:**

Laparoscopic sleeve gastrectomy (LSG) is considered to be an effective procedure for patients with morbid obesity. Belching is frequently reported after this procedure, but it has not been well studied in the bariatric population. This study aims to assess the changes in belching before and after sleeve gastrectomy, as measured with impedance monitoring.

**Methods:**

In a prospective study, patients underwent 24-h pH-impedance monitoring before and 3 months after LSG. Using this technique, belches can be identified. Preoperative and postoperative upper gastrointestinal symptoms were assessed using the Reflux Disease Questionnaire (RDQ).

**Results:**

Fifteen patients (1 M/14 F, mean age 42.2 ± 11.0 years, mean weight 134.5 ± 21.1 kg, mean BMI 46.4 ± 6.0 kg/m^2^) participated in this study. Belching occurred significantly more often after LSG, with an increase in symptom score from 2.9 ± 2.6 before to 5.3 ± 3.5 3 months after LSG (*p* = 0.04). The total number of gastric belches increased from 29.7 ± 11.7 before to 59.5 ± 38.3/24 h 3 months after LSG (*p* = 0.03). The total number of supragastric belches did not change after LSG. The number of swallows decreased from 746.9 ± 302.4 before to 555.7 ± 172.5 3 months after the procedure (*p* = 0.03). The number of air swallows tended to decrease (*p* = 0.08).

Esophageal acid exposure increased significantly, from 3.7 ± 2.9 % before to 12.6 ± 10.5 % after LSG (*p* = 0.01).

**Conclusion:**

Subjectively (as reported by patients) and objectively (as measured by impedance monitoring), an increase in gastric belches is seen after LSG, while the number of (air) swallows tends to decrease after the procedure and the incidence of supragastric belches remains constant. The altered anatomy as well as increased gastroesophageal reflux after LSG may play a role in the increase of belching.

## Introduction

Laparoscopic sleeve gastrectomy (LSG) has been shown to be an effective procedure for patients with morbid obesity [1]. The reduced gastric capacity as a result of this procedure leads to a decrease in the size of meals and earlier satiety, thus inducing weight loss. We noticed that troublesome belching is a frequently reported symptom by patients after LSG, especially in the first half year postoperatively.

Belching is a physiological mechanism that serves to vent ingested air from the stomach. During each swallow, a small amount of air is ingested [2]. Accumulation of air causes distention of the proximal stomach, which elicits a vagally mediated reflex resulting in transient relaxation of the lower esophageal sphincter (TLESR) [3,4]. During these TLESRs, the trapped intragastric air is vented from the stomach. Apart from being a physiological phenomenon, belching can also be a bothersome symptom, sometimes in isolation, but often associated with conditions such as gastroesophageal reflux disease (GERD) and functional dyspepsia [5]. Patients with GERD swallow more often, probably to clear the acid from the esophagus [6], and esophageal acid exposure is significantly increased after LSG [7]. It is thus conceivable that increased belching after LSG is reflux-associated.

However, in addition to the “classical” gastric belching referred to above, a second type of belching exists which is known as supragastric belching. In this type, the eructated air is not of gastric origin but is sucked into the esophagus less than a second before its expulsion [8]. Patients complaining of excessive belching almost invariably suffer from an excess of supragastric, and not of gastric belching, and in a subset of patients with GERD, increased supragastric belching has been observed [9,10]. Furthermore, it has been reported that belching patterns (gastric and supragastric) can be affected differentially by upper gastrointestinal surgery, such as fundoplication, which decreases the incidence of gastric belches but increases supragastric belching [11].

We hypothesized that gastric belching occurs more often after LSG due to the altered anatomy and—as a consequence—increased reflux. We therefore evaluated changes in belching before and after sleeve LSG, as observed by patients having undergone the procedure and measured by 24-h pH-impedance monitoring.

## Methods

### Study Design

In a prospective study design, 15 obese patients were included. GERD symptoms were recorded preoperatively, as described below, but these were not used as criteria for inclusion or exclusion. All patients underwent LSG at the Catharina Hospital in Eindhoven, The Netherlands. Before and 3 months after the procedure, symptoms of belching were evaluated using validated questionnaires, and 24-h pH-impedance monitoring and high-resolution manometry of the esophagus were performed. Upper gastrointestinal endoscopy and barium esophagogram were performed upon indication only. After sleeve gastrectomy, a proton pump inhibitor (PPI) was prescribed to all patients for the first 3 months.

### Procedures

#### Surgical Technique

Using a laparoscopic approach, a tubular gastric pouch of 75–120 mL was created by inserting a bougie (34 French) along the lesser curvature of the stomach. The pouch was created using a stapler starting about 6 cm proximal to the pylorus and continuing parallel to the lesser curvature of the stomach to the angle of His ending approximately 1 cm to the left of the esophagus [12].

#### Questionnaire

Before the procedure, all patients completed the validated Reflux Disease Questionnaire (RDQ) [13]. The RDQ consists of 22 questions: 11 assessing the frequency and 11 assessing the severity of symptoms. Questions in the RDQ assess the frequency and severity of heartburn, regurgitation, epigastric pain, belching, dysphagia, odynophagia, nausea, and vomiting. The frequency can be scored as no symptoms to daily symptoms and the severity from no burden to severe symptoms. Three months postoperatively, the symptoms were reassessed with the RDQ.

#### 24-h Multichannel Intraluminal Impedance pH Monitoring

Patients had to stop their PPI therapy 72 h before the measurement. It was performed using a single catheter with one inbuilt pH electrode and an array of impedance electrodes (Unisensor AG, Attikon, Switzerland). After calibration of the pH electrode in buffers of pH 4.01 and 7.01, the catheter was introduced transnasally and positioned with the pH sensor 5 cm above the proximal border of the lower esophageal sphincter (LES). The position of the LES was assessed manometrically before insertion of the pH-impedance catheter.

Impedance recording segments were located at 2–4, 4–6, 6–8, 8–10, 14–16, and 16–18 cm above the upper border of the LES. A sample frequency of 50 Hz was used to record impedance signals. The catheter was connected to a portable data logger (Ohmega, MMS, Enschede, The Netherlands).

During esophageal impedance monitoring, the resistance to electrical flow in an alternating current circuit is measured. This circuit is generated between two ring electrodes separated by a nonconductive catheter in the esophagus. Impedance is inversely associated with the conductivity of the medium surrounding the two electrodes. The conductivity of air is almost infinitively low, and thus, a high impedance is measured when the medium consists of air, while the conductivity of liquids (such as saline or gastric juice) is high and impedance low when these substances form the medium surrounding the electrodes. When placing a series of electrodes along an esophageal catheter, one can determine the movements of liquids and gas in the esophagus [14].

In patients with symptoms of belching, the nature of the belches can be determined. Two different types can be distinguished, i.e., the *gastric* belch, which is the consequence of physiologic venting of gastric air and the *supragastric* belch, characterized by eructation of esophageal air that was brought into the esophagus by suction or pharyngeal injection immediately before the belch [15]. In supragastric belches, the air has not reached the stomach before it is expelled.

Patients were instructed to fill in a standardized diary. They were encouraged to engage in their usual daily activities. The meals and beverages were recorded, as well as the moments when patients experienced symptoms, using the event marker button.

#### Data Analysis

**Fig. 1:**
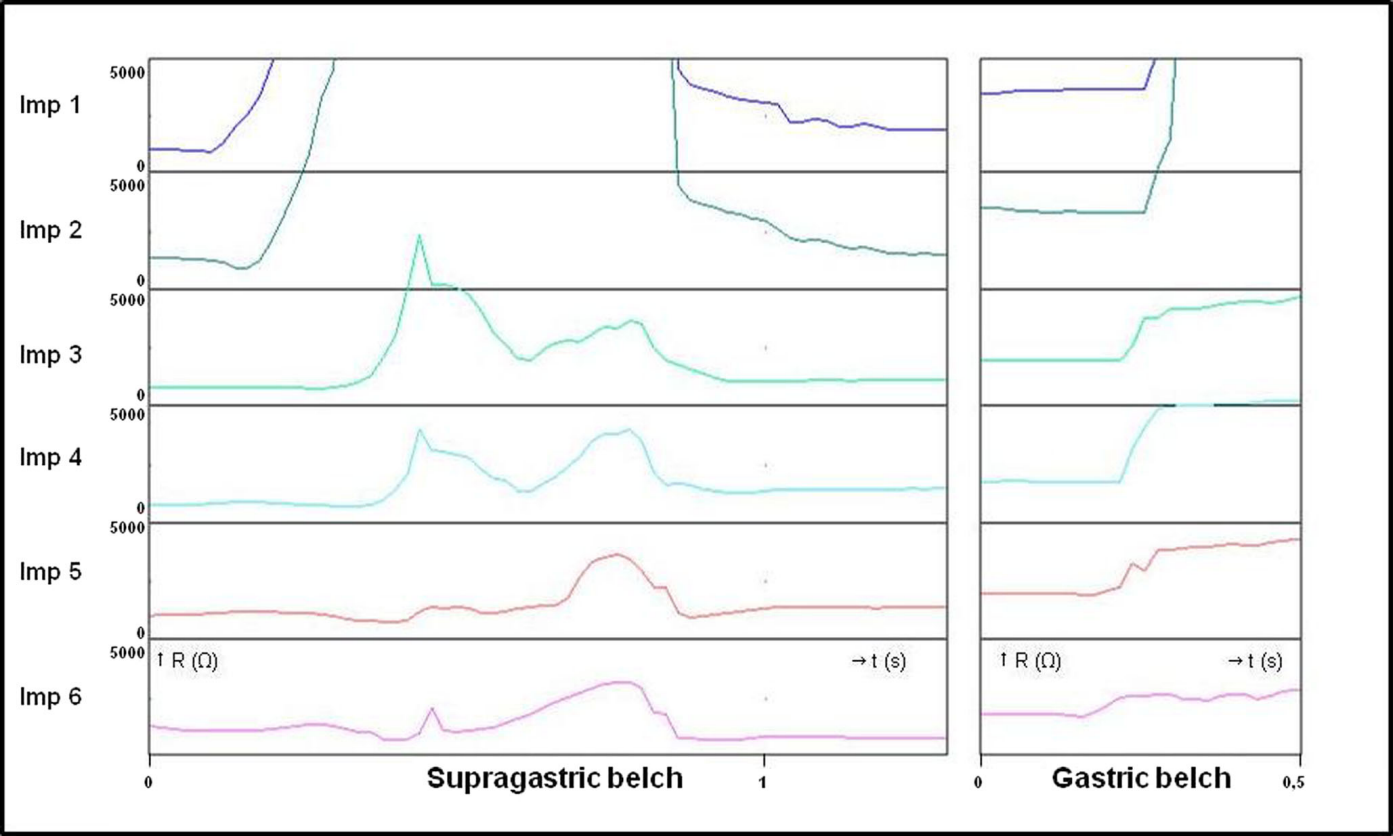
Gastric belch and supragastric belch

All 24-h impedance tracings were analyzed manually. Gas-containing reflux episodes (pure gas and mixed liquid/gas reflux episodes) reaching the most proximal impedance recording segment were regarded as *gastric* belches [16] (Fig. 1).

The registration periods of the patients were not exactly 24 h, because meal periods were excluded from the analysis. Therefore—to make the registrations comparable—all the registrations were extrapolated to 24 h, and the upright and supine periods were standardized to 16 and 8 h, respectively.

A *supragastric* belch was defined as a rapid rise in impedance (≥1000 Ω) moving in an aboral direction, followed by a return to baseline moving in the opposite direction (Fig. 1). Belches were considered to be related to reflux when a supragastric or gastric belch occurred immediately (<1 s) before the onset of a reflux episode or when the (supra)gastric belch occurred during the reflux episode [8].

**Fig. 2:**
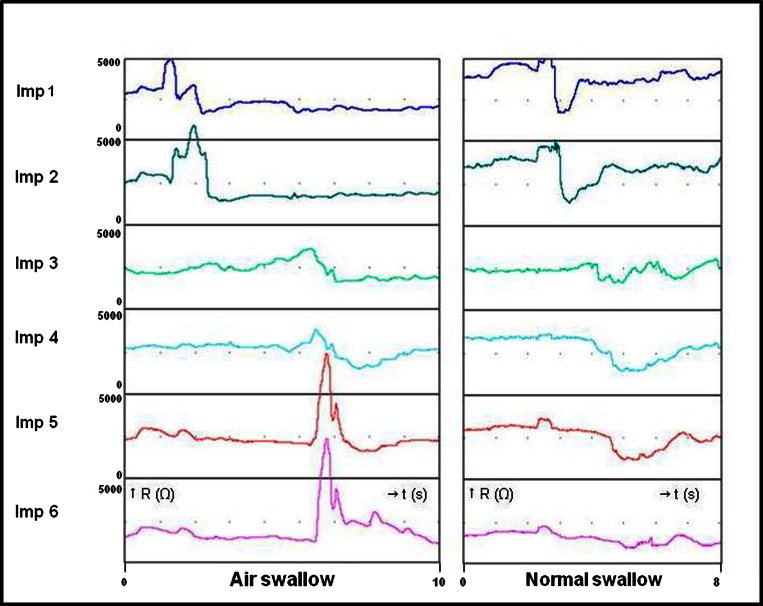
Air swallow and normal swallow

*Swallows* were defined as a fall in impedance from the most proximal to the most distal recording segment. *Air swallows* were distinguished as swallows accompanied by a gaseous component, characterized by a distally propagated rise in impedance reaching a value ≥1000 Ω in the most distal recording segment [17] (Fig. 2).

### Statistical Analysis

The statistical analysis was performed using SPSS version 20 (SPSS, Chicago, IL, USA). For comparison of means, the Wilcoxon signed-rank test was used. Data were presented as mean and standard deviation (SD), unless indicated otherwise. A two-sided *p* value of 0.05 was considered statistically significant. Reflux and belching events were identified automatically by the analysis software (MMS, Enschede, The Netherlands) but visually verified by the first author.

## Results

*Baseline Characteristics.* Fifteen patients (1 M/14 F, mean age 42.2 ± 11.0 years, mean weight 134.5 ± 21.1 kg, mean BMI 46.4 ± 6.0 kg/m^2^) were included in this study (Table 1). Five of the 15 patients used a PPI before surgery. Nine of the 15 patients had a hiatal hernia, as evidenced by the presence of a double high-pressure zone at high-resolution manometry.

**Table 1:** Baseline characteristics

Number of patients	15
Sex	1 M/14 F
Age (years)	42.2 ± 11.0
Weight (kg)	134.5 ± 21.1
BMI (kg/m^2^)	46.4 ± 6.0

Data expressed as mean ± SD

*Symptoms* There was a trend toward a higher total RDQ symptom score after the procedure (20.1 ± 17.3 before vs. 27.4 ± 15.8 3 months after LSG (*p* = 0.07)). The belching score increased significantly from 2.9 ± 2.6 before to 5.3 ± 3.5 3 months after LSG (*p* = 0.04). Following the LSG, 11 patients reported belching symptoms more frequently, 3 patients reported them less frequently, and 1 patient reported no difference. The severity of the symptoms was reported to have been increased in 10 patients, 2 patients reported less severe symptoms and 3 patients reported no difference.

*Belches* The total number of gastric belches per 24 h increased from 29.7 ± 11.7 before to 59.5 ± 38.3 3 months after LSG (*p* = 0.03, see Table 2). This was mainly caused by an increase in gastric belches not associated with a reflux episode (“gas only”) (from 11.0 ± 6.7 before to 34.7 ± 26.7 3 months after LSG (*p* = 0.004)). Mixed liquid/gas reflux events increased from 18.6 ± 9.8 before to 24.6 ± 18.6 after LSG (*p* = 0.43). The number of supragastric belches was not altered by LSG (12.6 ± 17.3 before and 8.7 ± 13.4 3 months after LSG (*p* = 0.73)).

**Table 2:** Gastric and supragastric belching before and 3 months after LSG

	Before LSG	3 months after LSG	*p* Value
Gastric belches (number/24 h)			
- Gas only	11.0 ± 6.7	34.7 ± 26.7	0.004
- Mixed liquid/gas reflux	18.6 ± 9.8	24.6 ± 18.6	0.43
Total number of gastric belches	29.7 ± 11.7	59.5 ± 38.3	0.03
Supragastric belches (number/24 h)			
- Gas only	6.2 ± 11.1	3.1 ± 4.0	0.67
- Mixed liquid/gas reflux	6.2 ± 7.5	5.6 ± 9.6	0.86
Total number of supragastric belches	12.6 ± 17.3	8.7 ± 13.4	0.73

Data expressed as mean ± SD

*Swallows* The total number of swallows significantly decreased from 746.9 ± 302.4 before to 555.7 ± 172.5 after LSG (*p* = 0.03). There was a trend toward a decrease in the number of air swallows (from 150.5 ± 95.2 before to 130.0 ± 76.3 after LSG (*p* = 0.08)) (Table 3).

**Table 3:** Swallows and air swallows before and 3 months after LSG

	Before LSG	3 months after LSG	*p* Value
Swallows			
- In upright position (16 h)	672.4 ± 278.5	499.3 ± 154.2	0.03
- In supine position (8 h)	74.6 ± 32.7	56.4 ± 27.6	0.03
Total number/24 h)	746.9 ± 302.4	555.7 ± 172.5	0.03
Air swallows			
- In upright position (16 h)	137.2 ± 92.6	123.0 ± 73.2	0.19
- In supine position (8 h)	13.3 ± 9.2	7.0 ± 5.4	0.02
Total number/24 h	150.5 ± 95.2	130.0 ± 76.3	0.08

Data expressed as mean ± SD

*Acid Reflux* The esophageal acid exposure significantly increased when comparing 24-h pH measurements before and 3 months after sleeve gastrectomy in these patients: upright reflux increased from 5.1 ± 4.5 % before to 13.0 ± 9.9 % (*p* = 0.01), supine reflux from 1.4 ± 2.5 to 11.4 ± 15.3 % (*p* = 0.02), and total reflux from 3.7 ± 2.9 to 12.6 ± 10.5 % (*p* = 0.01) 3 months after LSG.

## Discussion

This study focused on belching, an upper gastrointestinal symptom often reported by patients after LSG. Our study confirmed the increase in patient-reported frequency and severity of belching after LSG. Furthermore, preoperative and postoperative esophageal impedance monitoring showed a significant increase in the number of gastric belches after sleeve gastrectomy. Supragastric belching was not affected by LSG.

It is tempting to speculate on the mechanisms by which LSG leads to increased gastric belching. Earlier studies have shown a decrease in resting LES pressure after LSG [7,18], although Petersen et al. reported an increased LES pressure [19]. Since the main mechanism involved in the pathogenesis of gastric belches is TLESR while the incidence of TLESRs is not related to LES pressure, it is unlikely that a change in basal LES pressure is responsible for the observed increase in belching.

Yehoshua et al. have reported that patients with a sleeve gastrectomy had a notably higher pressure in the sleeve, reflecting a reduced distensibility compared to that of the whole stomach and the removed fundus of the stomach [20]. Since the main stimulus for TLESRs is distension of the proximal stomach, it seems plausible that the observed increase in gastric belches is the consequence of an increased incidence of TLESRs. Another possibility is that after LSG belches are not TLESR-mediated but occur as a consequence of an increased intragastric pressure caused by Boyle’s law (pressure of gas increases when volume decreases).

Another explanation for the observed changes may be that the increased belching is a consequence of the increased esophageal acid exposure after sleeve gastrectomy, since reflux episodes are often accompanied by belches (mixed liquid/gas reflux) [21]. Indeed, our study confirmed a previous observation that LSG increases gastroesophageal reflux [7]. However, in our study, the gas only belches markedly increased but the mixed liquid/gas reflux events did not, suggesting that the increased belching after LSG cannot simply be explained as a consequence of increased reflux.

As discussed before, there is an association between the number of swallows and the number of belches [21]. Our study showed an increase in belching after sleeve gastrectomy but a decrease in swallowing. This suggests that the increase in belching is a consequence of the sleeve gastrectomy itself and not of the rate of swallowing.

Concerning other bariatric procedures, there is limited information available in current literature. De Jong et al. reported an increase of belching after gastric banding [22]. Bellam et al. described an improvement of belching after gastric bypass surgery [23]; however, all these studies only reported on symptoms reported by the patient and not by objective measurements.

This study has some limitations: The number of included patients is relatively small. Furthermore, the patients were measured 3 months after the operation. We have the impression that over time, belching symptoms decrease, although this has not been confirmed by objective measurements yet.

In conclusion, patients report more belching symptoms after LSG. Impedance monitoring shows an increase of gastric belches after sleeve gastrectomy although the number of (air) swallows appears to decrease after sleeve gastrectomy. Further studies—especially into the incidence of TLESRs before and after sleeve gastrectomy—are needed to unravel the exact pathophysiology of the increased frequency of belching following LSG and their pattern over time.
